# Systematic Review and Meta-Analysis of the Association between Complement Factor H I62V Polymorphism and Risk of Polypoidal Choroidal Vasculopathy in Asian Populations

**DOI:** 10.1371/journal.pone.0088324

**Published:** 2014-02-10

**Authors:** Zhao-Yang Wang, Keke Zhao, Jingwei Zheng, Brian Rossmiller, Cristhian Ildefonso, Manas Biswal, Pei-quan Zhao

**Affiliations:** 1 Department of Ophthalmology, Xinhua Hospital, Shanghai Jiaotong University School of Medicine, Shanghai, China; 2 Departments of Molecular Genetics, University of Florida, Gainesville, Florida, United States of America; 3 Eye Hospital, Wenzhou Medical University, Wenzhou, Zhejiang, China; University of Iowa, United States of America

## Abstract

**Purpose:**

To investigate whether the polymorphism rs800292 (184G>A, I62V) in the complement factor H gene is associated with polypoidal choroidal vasculopathy (PCV) and the genetic difference between PCV and neovascular age-related macular degeneration (nAMD), in Asian populations.

**Methods:**

A comprehensive literature search was performed in PubMed, Medline, Web of Science, and reference lists. A system review and meta-analysis of the association between I62V and PCV and/or nAMD were performed from 8 studies involving 5,062 subjects. The following data from individual studies were extracted and analyzed: 1) comparison of I62V polymorphisms between PCV and controls; 2) comparison of I62V polymorphisms between PCV and nAMD. Summary odds ratios (ORs) and 95% confidence intervals (CIs) were estimated using fixed-effects models. The Q-statistic test was used to assess heterogeneity, and Egger’s test was used to evaluate publication bias. Sensitivity analysis and cumulative meta-analysis were also performed.

**Results:**

The I62V polymorphism showed a significant summary OR_1_ for genotype GA+GG versus homozygous genotype AA was 3.18 (95% CI, 2.51–4.04, *P*<*0.00001*), the OR_2_ of heterozygous genotype GA versus AA was 2.29 (95% CI: 1.79–2.94, *P*<*0.00001*), the OR_3_ of homozygous genotype GG versus AA was 4.42 (95% CI: 3.45–5.67, *P*<*0.00001*), and the OR_4_ of allele G versus A was 2.04 (95% CI: 1.85–2.26, *P*<*0.00001*). Sensitivity analysis indicated the robustness of our findings, and evidence of publication bias was not observed in our meta-analysis. Cumulative meta-analysis revealed that the summary ORs were stable. There was no significant difference in every genetic model between PCV and nAMD (n = 5, OR_1_ = 0.92, OR_2_ = 0.96, OR_3_ = 0.90, OR_4_ = 0.94).

**Conclusions:**

Our analysis provides evidence that the I62V polymorphism is associated with an increased risk of PCV. The variant of I62V could be a promising genetic biomarker of PCV in Asian populations.

## Introduction

Polypoidal choroidal vasculopathy (PCV) is a hemorrhagic and exudative macular disorder that is characterized by inner branching choroidal networks with surrounding polypoidal dilatation of the choroidal vessels, which can be clearly demonstrated by indocyanine green angiography [1].

PCV can occur in any gender or race, but it is more commonly seen in Asians than in Caucasians, accounting for 24.5% of patients with findings suggestive of neovascular age-related macular degeneration (nAMD) in the Chinese population [2], for 24.6% in the Korean population [3], for 54.7% in the Japanese population [4], but for only about 4–9.8% in Caucasians. [1].

PCV is categorized by some experts as a subtype of nAMD, [4], [5] but others consider it as a different disease entirely. [3], [6]–[8] Clinically, PCV shares several common manifestations with nAMD, such as subretinal exudation and hemorrhage involving the macular region. However, important differences have been noted that patients with PCV are younger and more likely Asians. Their eyes are lack of drusen, often occurrence with serosanguinous maculopathy or hemorrhagic pigment epithelial detachment. They show different responses to photodynamic therapy and therapy involving anti-vascular endothelial growth factor (VEGF) agents.

There are also significant differences in angiographic and optical coherence tomography features between PCV and nAMD. Histopathological studies suggest differences in the anatomical details of the associated vascular abnormalities in the retina and choroids and the relative role of VEGF. These similarities and differences have been a subject of much interest and debate regarding whether the vascular abnormality in PCV represents neovascularization or a phenotype distinct from choroidal neovascularization (CNV). [9].

The etiology of PCV remains largely unknown. It is known as a multifactorial disease due to multiple environmental risk factors and genetic factors or to the interactions between these. The phenotypic similarities between PCV and nAMD lead to the hypothesis that genes involved in AMD may also play a role in PCV. [9] Therefore, investigators have now focused on comparing these two entities to discover if these two different phenotypes can be attributed to genetic differences that may reveal different underlying pathogenic mechanisms. Several candidate genes such as complement factor H (CFH), high temperature required factor A1 (HTRA1), and age-related maculopathy susceptibility 2 (ARMS2) have been reported to increase the risk of AMD and PCV development. [10]–[12] The CFH I62V coding variant (rs800292) on chromosome 1q32 has been extensively studied via genetic and molecular approaches, which provide strong statistical evidence for disease association and a plausible biologic context supporting this variant as an attractive candidate for a causal polymorphism leading to the development of AMD and PCV. [13]–[15] However, the population heterogeneity and relatively small sizes studies warrant confirmation of the association of I62V with PCV across different studies in different populations. Here we conducted a meta-analysis of previous studies representing an assessment of the association between the CFH I62V variant and PCV and/or nAMD, comprising a total of 5,062 subjects, to more reliably compare the genetic effect of CFH I62V between PCV and nAMD.

## Materials and Methods

### Identification and Eligibility of Relevant Studies

This meta-analysis was conducted according to the PRISMA guidelines. [16] We searched PubMed, Medline and Web of Science using the following search terms: (“polypoidal choroidal vasculopathy” OR “PCV”) and (“CFH” OR “complement factor H”), and other alternative names (I62V, Val62Ile,184G>A, rs800292). All related articles should have been published before Aug 31, 2013 and without any language limitation.

Studies were included only if they fulfill all of the following five criteria: (1) All patients had a complete ophthalmic examination, including fundus photography, and fluorescent or indocyanine green angiography (ICG). The diagnostic criteria of PCV was based on these clinical features and ICG showing a branching vascular network terminating in polypoidal swelling. The diagnostic criteria of nAMD based on the clinical features and grading were classified using a standard grid suggested by the International Age-related Maculopathy Epidemiologic Study Group for age-related maculopathy. (2) Study design was limited to case-control study, cohort study, or population-based epidemiologic survey, not a review, case report, or editorial comment. (3) The major study objective was to evaluate the association between CFH I62V polymorphism and PCV and/or nAMD. (4) Raw data of allele or genotype frequencies or counts available. Allele was A/G, and the genotypes covered AA, AG, and GG. (5) For studies published by the same group on the same gene and markers, only the most recent report or the report with the largest sample size was included for analysis.

### Data Extraction

Two reviewers (Z.Y.W. and K.K.Z.) independently extracted the data and evaluated the quality. The following variables were extracted from each study: the name of the first author, year of publication, ethnicity, phenotype of cases evaluated, sample size, mean age and sex ratio of study participants, and allele and genotype distributions in cases and controls. If publications listed allele and genotype counts stratified according to the PCV sub-phenotype, they were combined into one case group.

Independent review and resolution by a third reviewer (P.Q.Z.) was sought if the two reviewers disagreed.

### Statistical Analysis

Hardy-Weinberg Equilibrium (HWE) was tested by the exact test to compare the observed genotype frequencies with the expected genotype frequencies within the control subjects. [17] To investigate the associations of I62V polymorphism with PCV, the allele and genotype frequencies of the SNPs between PCV and controls were compared. To determine whether PCV and nAMD have different genetic risks, the allele frequencies of the SNPs were compared between patients with PCV and nAMD in the studies that included both disorders. The following four odds ratios (ORs) and their 95% confidence intervals (95% CIs) were calculated in each study: OR_1_ for (GG+GA) versus AA, OR_2_ for GA versus AA, OR_3_ for GG versus AA, and OR_4_ for allele G versus A.

Between-study heterogeneity was assessed by the Q-statistic test and *I^2^* statistic. [18], [19] A *P* value <0.1 was considered statistically significant for the Q-statistic test. [18] *I^2^* ranges between 0% and 100% (where a value of 0% represents no heterogeneity) and larger values represent increasing heterogeneity. The fixed-effects estimates were described in the text for originally homogenous or post hoc homogenized datasets. If there was evidence of between-study heterogeneity, random-effects estimates were described. [18], [19].

To assess the publication bias and small-study bias, a funnel plot of the data was applied. In addition, Egger’s test was used to detect publication bias. [20] A leave-one-out sensitivity analysis was performed by iteratively removing 1 study at a time to confirm that our findings were not driven by any single study. Cumulative meta-analysis was performed to evaluate the accumulation of evidence on the association between CFH I62V and PCV. The results of individual studies were pooled using the software Review Manager (version 5.2, the Cochrane Collaboration, Oxford, England; available at: http://ims.cochrane.org/revman. Accessed June 30, 2013). All other statistical analysises were performed using Stata software (version 11.0; Stata Corporation, College Station, TX). All tests were 2-tailed. A *P* value <0.05 was considered statistically significant except for the test of between-study heterogeneity.

## Results

### Eligibility of Studies

**Figure 1:**
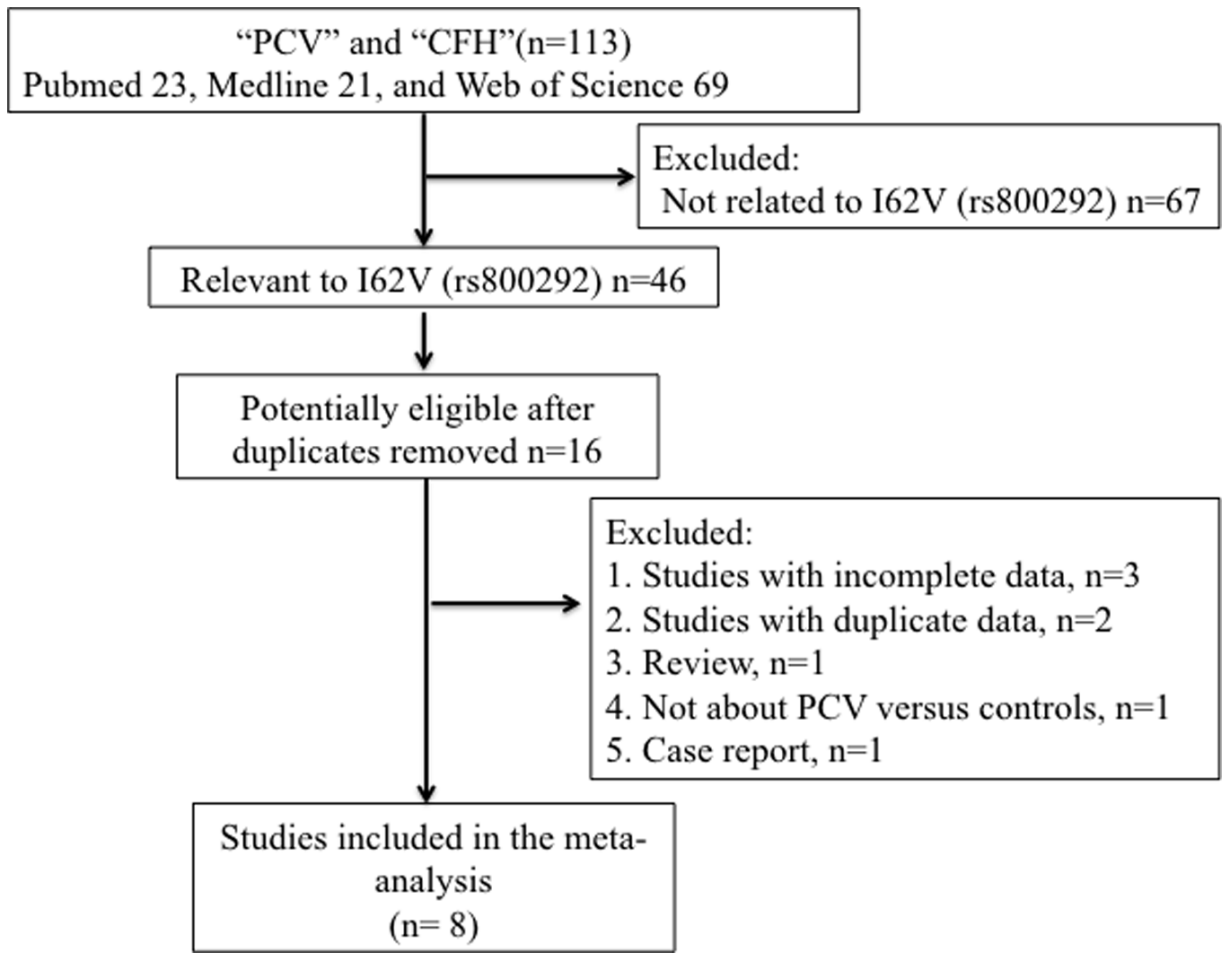
The literature search process. Flow diagram depicts the screening process of retrieved articles, including the reason for and number of exclusions. CFH = complement factor H; PCV = polypoidal choroidal vasculopathy.

A total of 113 relevant studies were identified by our initial search, of which 8 studies were eligible for inclusion in the review. [21]–[28] Two of these studies did not have genotype information, but authors kindly provided supplementary information. [22], [23] Figure 1 shows the flow chart of the selection process used to identify the studies concerned. Table 1 lists the studies included in the meta-analysis together with summary characteristics of study subjects. The combined sample size for this meta-analysis was 5,062, which included 1,680 PCV patients, 1,015 nAMD patients and 2,367 controls. The average ages ranged from 63.8 to 75.7 years in PCV groups, 67.0 to 77.7 years in nAMD groups and 51.2 to 72.2 years in control groups. Gender ratios (male/female) in the 3 groups varied from 0.98 (48/49) to 5.28 (147/28) in PCV groups, 1.80 (101/56) to 3.62 (76/21) in nAMD groups, and from 0.67 (111/166 ) to 3.41 (116/34) in control groups. All studies, except 1 study [22] was cohort design, were case–control designs with subjects of Asian ancestry, 5 studies were conducted in Japan, 1 study was conducted in China, 1 study was conducted in Singapore, 1 study was conducted in Thailand (Table 1).

**Table 1:** Main Characteristics of the Studies Included in the Meta-Analysis.

First Author	PublishedYear	Ethnic	Totlal (N)			Average Age(yrs)			Gender Ratio(M/F)			StudyType
			PCV	nAMD	Control	PCV	nAMD	Control	PCV	nAMD	Control	
Miki^21^	2013	Japanese	175	NA	150	74.2±7.572.1±7.1	NA	72.1±5.6	147/28	NA	116/34	Casecontrol
Zhang^22^	2013	Chinese	250	157	204	65±8.6	67±9.2	69±9.0	166/84	101/56	124/80	Cohort
Ueda-Arakawa^23^	2013	Japanese	87*	97*	NA	71.5±8.4	74.8±8.3	NA	70/17	76/21	NA	Casecontrol
Chantaren^24^	2012	Thai	97	NA	102	62.9±8.9	NA	62.2±7.6	48/49	NA	50/52	Casecontrol
Tanaka^25^	2011	Japanese	381	253	277	69.9±.9.1	73.7±7.5	72.9±8.7	271/110	188/65	111/166	Casecontrol
Hayashi^26^	2010	Japanese	518*	408*	1351*	75.1±8.5	77.7±8.4	51.2±16.3	381/137	293/115	722/629	Casecontrol
Goto^27^	2009	Japanese	100*	100*	190*	72.7±8.3	74.6±8.8	72.2±8.5	81/19	73/27	86/104	Casecontrol
Lee^28^	2008	SingaporeChinese	72	NA	93	63.8±7.6	NA	67.2±4.6	46/26	NA	40/53	Casecontrol

NA = not available; nAMD = neovascular age-related macular degeneration; PCV = polypoidal choroidal vasculopathy.

*The number of cases or controls was changed in the next meta-analysis, but the reason was not given.

### Quantitative Synthesis

Allele and genotype distributions for the I62V polymorphism from individual studies are shown in Table 2. Except one study not observing HWE [22], and one study without a control group [23], all other studies observed HWE and were included in pooling (Table 2).

**Table 2:** Allele and Genotype Distribution of the I62V Polymorphism.

FirstAuthor	PCV	Genotype				GAllele	nAMD	Genotype				GAllele	Control	Genotype				GAllele	HWE
	(N)	GG	GA	AA	A/G	Frequency(%)	(N)	GG	GA	AA	A/G	Frequency(%)	(N)	GG	GA	AA	A/G	Frequency(%)	P value
Miki^21^	175	100	65	10	85/265	75.71	NA	NA	NA	NA	NA	NA	150	54	63	33	129/171	57.00	0.08
Zhang^22^	250	131	107	12	131/369	73.80	157	92	52	13	78/236	75.16	204	75	85	44	173/235	57.60	0.04
Ueda-Arakawa^23^	80	51	22	7	36/124	77.50	87	56	25	6	37/137	78.74	NA	NA	NA	NA	NA	NA	NA
Chantaren^24^	97	46	47	4	55/139	71.65	NA	NA	NA	NA	NA	NA	102	29	59	14	87/117	57.35	0.06
Tanaka^25^	381	210	150	21	192/570	74.80	253	147	93	13	119/387	76.48	277	100	141	36	213/341	61.55	0.21
Hayashi^26^	511	290	182	39	260/762	74.56	400	228	148	24	196/604	75.50	1338	456	649	233	1115/1561	58.33	0.94
Goto^27^	95	47	43	5	53/137	72.11	96	47	46	3	52/140	72.92	188	60	92	36	212/164	56.38	0.94
Lee^28^	72	41	27	4	35/109	75.69	NA	NA	NA	NA	NA	NA	93	36	41	16	73/113	60.75	0.47

HWE = HardyeWeinberg equilibrium; NA = not available; nAMD = neovascular age-related macular degeneration; PCV = polypoidal choroidal vasculopathy.

**Figure 2:**
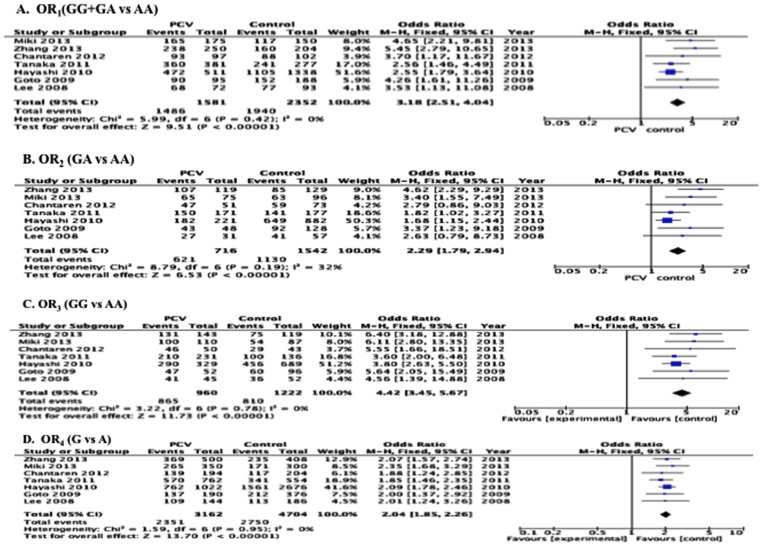
Forest plots of meta-analysis of the association between I62V polymorphism and PCV. Odds ratios (black *squares)* and 95% confidence intervals (bars) are given for each study. Also shown are the *diamonds* of the summary ORs based on the Mantel–Haenszel fixed-effects model (M-H Overall). CI = confidence interval; OR = odds ratio. A: OR_1_(GG+GA vs AA); B: OR_2_ (GA vs AA); C: OR_3_ (GG vs AA); D: OR_4_ (G vs A).

We initially performed a meta-analysis based on different genetic models between PCV and Control groups. In the fixed-effects model, the pooled OR_1_ for the risk allele (GA+GG) versus AA was 3.18 (95% CI, 2.51–4.04, *P*<*0.00001*), pooled OR_2_ for GA versus AA was 2.29 (95% CI: 1.79–2.94, *P*<*0.00001*), pooled OR_3_ for GG versus AA was 4.42 (95% CI: 3.45–5.67, *P*<*0.00001*), and pooled OR_4_ for the risk allele G versus A was 2.04 (95% CI: 1.85–2.26, *P*<*0.00001*) (Figure 2). The heterogeneity tests of these 4 comparisons are also shown in Figure 2.

We also calculated four summary ORs between PCV group and nAMD group. No significant difference between PCV and nAMD (n = 5, OR_1_ = 0.92, OR_2_ = 0.96, OR_3_ = 0.90, OR_4_ = 0.94) was found in each genetic model (Table 3).

**Table 3:** Meta-analysis compared the allelic frequencies of I62V between PCV and nAMD.

		Sample Size			Test of Association	Test of Heterogeneity	
Polymorphism	Studies(N)	PCV	nAMD	Statistical Method	OR (95% CI)	P Value	I^2%^
(GA+GG) vs AA	5	1317	993	Odds Ratio (M-H, Fixed, 95% CI)	0.92 [0.65, 1.30]	0.48	0
GA vs AA	5	588	423	Odds Ratio (M-H, Fixed, 95% CI)	0.96 [0.67, 1.38]	0.27	22
GG vs AA	5	813	629	Odds Ratio (M-H, Fixed, 95% CI)	0.90 [0.63, 1.27]	0.69	0
G vs A	5	2634	1986	Odds Ratio (M-H, Fixed, 95% CI)	0.94 [0.82, 1.07]	1	0

CI = confidence interval; M-H = Mantel–Haenszel model; nAMD = neovascular age-related macular degeneration; OR = odds ratio; PCV = polypoidal choroidal vasculopathy.

### Sensitivity Analysis and Cumulative Meta-Analysis

**Figure 3:**
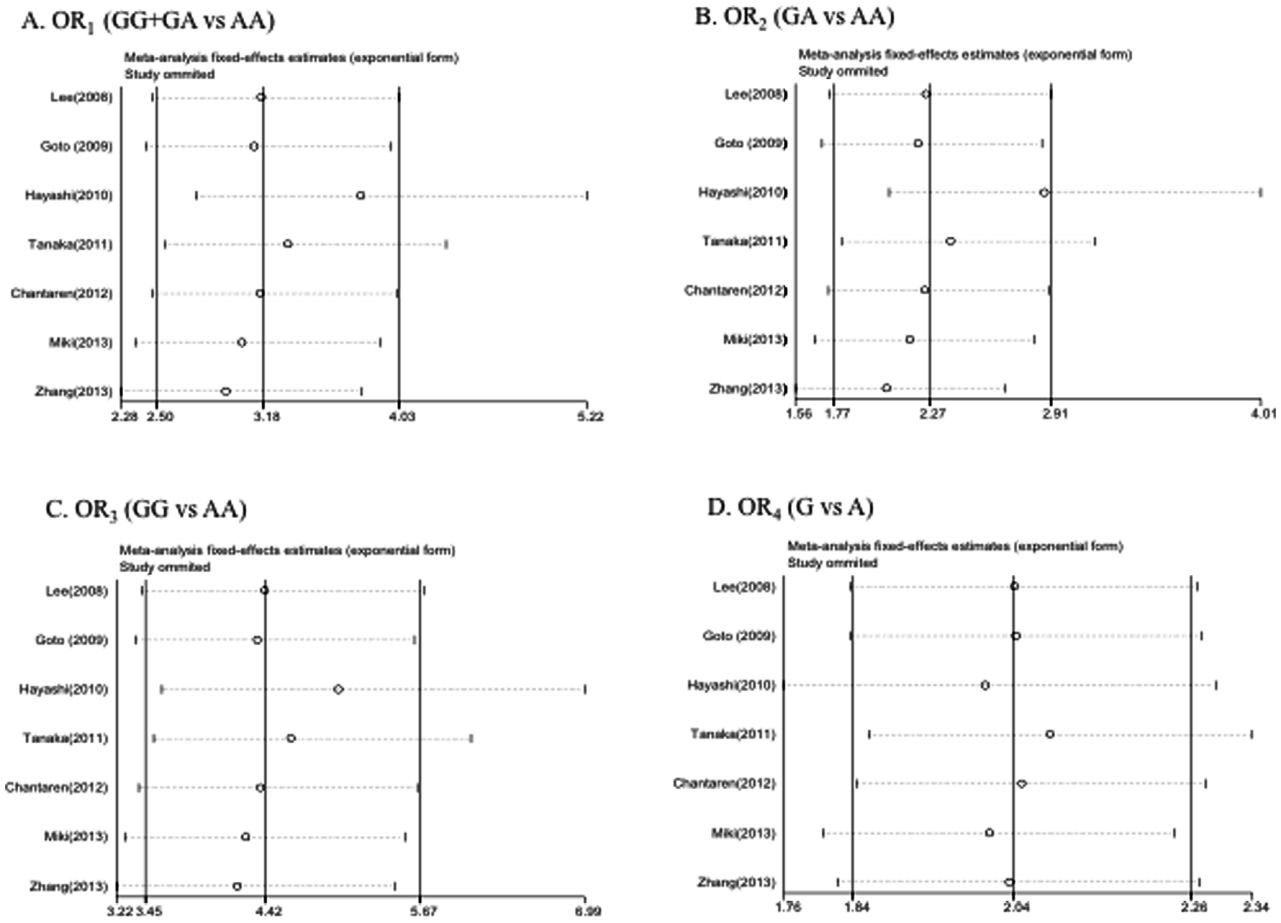
Results of Leave-One-Out Sensitivity Analysis. The horizontal axis shows the omitted study. The horizontal axis represents the odds ratio. Every circle indicates the pooled OR when the left study is omitted in this meta-analysis. The two ends of every broken line represent the respective 95% confidence interval. A: OR_1_(GG+GA vs AA); B: OR_2_ (GA vs AA); C: OR_3_ (GG vs AA); D: OR_4_ (G vs A).

To evaluate the robustness of the association results, we performed a leave-one-out sensitivity analysis by iteratively removing one study at a time and recalculating the summary OR. The summary ORs remained stable (Figure 3), indicating that our results were not driven by any single study and that similar results could be obtained after excluding the two study not observing the HWE.

**Figure 4:**
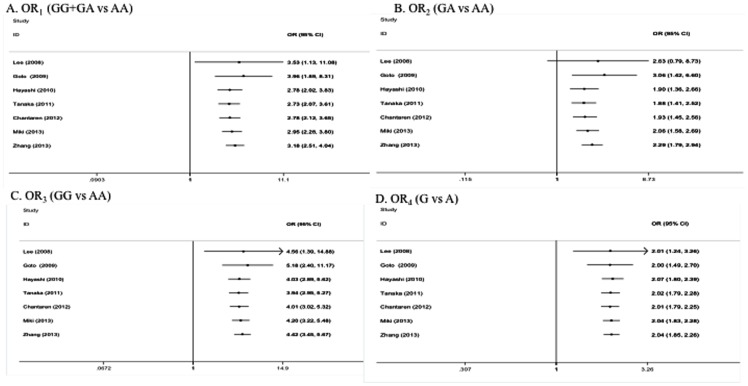
Cumulative meta-analysis of the association between I62V polymorphism and PCV. Every circle represents the pooled OR when studies accumulated over time, and the horizontal line represents the 95% confidence interval of pooled OR. A: OR_1_(GG+GA vs AA); B: OR_2_ (GA vs AA); C: OR_3_ (GG vs AA); D: OR_4_ (G vs A).

The cumulative meta-analysis revealed that the summary ORs were very high in the first two studies but did not vary much after the third study (Figure 4).

### Publication Bias

We assessed publication bias using Egger’s test. No statistically significant evidence of publication bias was detected for the OR_1_ (P = 0.12), OR_2_ (P = 0.10), OR_3_ (P = 0.11) and OR_4_ (P = 0.82).

## Discussion

The reliable assessment of the association between *CFH* I62V and PCV has been hindered in low frequency of variant alleles and small sample sizes in studies. To overcome these barriers, we performed a systematic meta-analysis to summarize the evidence to date regarding the association between CFH I62V and PCV, representing a pooled total of 6 case-control and 1 cohort studies between PCV and Control, 5 case-control studies between PCV and nAMD, involving 5,062 subjects.

Our meta-analysis focused on the association between the CFH I62V polymorphism and PCV risk specifically. The results indicate a strong association between CFH I62V and PCV with no evidence of publication bias. The ORs of all comparisons supported the view that I62V is a risk factor for PCV in Asian populations, and these results also showed that the G allele might be a PCV-causing locus and that the GG homozygote genotype (4.4-fold) had a stronger effect than the GA heterozygote genotype (2.3-fold) with a significant dose response correlation. In individuals carrying at least one copy of the risk allele, disease risk was increased by 3.2-fold.

In addition, sensitivity analyses by iteratively removing one study at a time including one study not observing HWE [22] and one study without control group [23] with the leave-one-out sensitivity analysis showed similar and consistent result, thus indicating the robustness of our findings.

CFH is a critical negative regulator of the alternative pathway of the complement system. It binds to C3b, promotes the decay of C3 convertase, and serves as a cofactor for the factor I–mediated proteolytic inactivation of C3b, resulting in the inhibition of the complement cascade. [29] The CFH gene is found on the 1q32 region. Although the pathogenetic mechanism of CFH leading to PCV is still unclear, the I62V coding variant (rs800292) in CFH has been extensively studied via genetic and molecular approaches, which provide strong statistical evidence for disease association and a plausible biologic context supporting this variant as an attractive candidate for a causal polymorphism leading to the development of AMD and PCV. [13]–[15] Recently, one meta-analysis, reported by Yuan et al [30], demonstrated that the I62V polymorphism is significantly associated with AMD in Asian populations but there is no link in Caucasian populations. In the current study, we provide evidence that the I62V polymorphism is associated with an increased risk of PCV. There was no significant difference in every genetic model between PCV and nAMD (n = 5, OR_1_ = 0.92, OR_2_ = 0.96, OR_3_ = 0.90, OR_4_ = 0.94) in our study.

Our meta-analysis did not found any heterogeneity in each of the OR. To minimize the bias of our research, we did not use the option of language limitation on PubMed, Medline, or Web of Science, and all previous studies that met our criteria were included. The study must be published in peer-reviewed journals; second, we collected the nAMD data in our studies, excluding the data of dry AMD and other type of wet AMD, such as Retinal angiomatous proliferation; third, all studies which fulfill the inclusion criteria were conducted with subjects of Asian ancestry, 5 studies were conducted in Japan, 1 study was conducted in China, 1 study was conducted in Singapore, 1 study was conducted in Thailand. We didn’t find any evidence or study about CFH I62V and PCV with subjects of Caucasian, Africa and other populations based upon our search strategy. Chen et al [31] also reported that the polymorphisms at CFH, LOC387715, HTRA1, and C2 were found to be significantly associated with PCV, which was similar to our conclusions. But in their research the number of included studies was 5, which were all included in our study.

Several limitations of this meta-analysis should be acknowledged. First, there were a limited number of original studies, and all reports were on Eastern Asians. There were no reports on research involving Caucasian, Africa or other populations. These conclusions remain to be confirmed by further research. Second, because the allele and genotype data were not available in several studies, [13], [32], [33] our meta-analysis may have not included all the studies that have been published. Third, by studying CFH I62V polymorphism and other CFH polymorphisms (e.g., Y402H, C3 and CFB) in this population, it may be useful to understand the effect of these variations on the onset and progression of PCV.

In conclusion, our analysis provides evidence that the CFH I62V polymorphism is associated with an increased risk of PCV in the Asian population. We found that Asian patients with the CFH I62V variant might have a higher risk of developing PCV compared with controls. Our results expand the number of confirmed PCV susceptibility loci for Asian populations and provide a better understanding of the genetic architecture underlying disease susceptibility. The potential for preclinical prediction in future genetic testing may advance by combined evaluation of inherited susceptibility with previously established loci. Further investigations are necessary to confirm the roles of the CFH I62V polymorphism reported in a limited number of original studies. Genetic analysis might provide timely preclinical prediction, prevention, and treatment for PCV.

## Supporting Information

Checklist S1 PRISMA checklist.
(DOC)

Flow Diagram S1 PRISMA Flow Diagram.
(DOC)
