# Supplementary material for: Systematic Review and Meta-Analysis of the Association between Complement Factor H I62V Polymorphism and Risk of Polypoidal Choroidal Vasculopathy in Asian Populations
Source: PLoS One. 2014 Feb 10;9(2):e88324. doi: 10.1371/journal.pone.0088324 (PMC3919738; doi:10.1371/journal.pone.0088324)
Supplement: Flow Diagram S1 — PRISMA Flow Diagram. (DOC) [file pone.0088324.s002.doc]

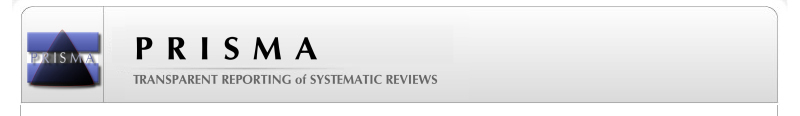
**PRISMA 2009 Flow Diagram**

**Screening**

**Included**

**Eligibility**

**Identification**

Records identified through database searching
(n = 113 )

Additional records identified through other sources
(n = 0 )

Records after duplicates removed
(n = 46 )

Records screened
(n = 46 )

Records excluded
(n =30 )

Full-text articles assessed for eligibility
(n = 16 )

Full-text articles excluded, with reasons
(n = 8 )

Studies included in qualitative synthesis
(n =8 )

Studies included in quantitative synthesis (meta-analysis)
(n = 8 )
